# Promoting college students’ systems thinking in asynchronous discussions: Encouraging students initiating questions

**DOI:** 10.3389/fpsyg.2022.1028655

**Published:** 2022-12-13

**Authors:** Yawen Yu, Gaowei Chen, Yang Tao, Xiaofeng Li, Lina Yang, Shengwen Dong

**Affiliations:** ^1^Faculty of Education, The University of Hong Kong, Hong Kong, Hong Kong SAR, China; ^2^Department of Library and Information Science, Pennsylvania Western University, Clarion, PA, United States; ^3^Graduate School, Tianjin Foreign Studies University, Tianjin, China; ^4^School of Nursing, Tianjin Medical University, Tianjin, China

**Keywords:** asynchronous discussions, systems thinking, network analysis, meaningful discourse, mixed method

## Abstract

**Introduction:**

Systems thinking is one of the most important thinking skills for medical students. Most of the studies focused on designing technological-rich learning environments which usually take several weeks or months to implement. However, the occurring of COVID-19 health crisis does not allow extensive period of time to implement classroom interventions. How to support students’ systems thinking in fully online environments remains an issue. This study examines if encouraging students initiating questions on asynchronous discussion forum supports their systems thinking development.

**Methods:**

Twenty-two junior students participated in this study. We compared if and how students developed systems thinking when they were encouraged asking questions in asynchronous discussion forums in one unit with another unit in which traditional method was used. Multiple analytical methods were applied in this study, including, social network analysis, epistemic network analysis, inferential statistical analysis and qualitative analysis.

**Results:**

Quantitative results showed that all students improved systems thinking compared with traditional teaching unit among which leader students improved most. Further analysis on students’ discussion posts suggested leader students asked high systems thinking level questions and provided high level responses. Epistemic network analysis unpacked how leader, regular and peripheral students engaged in initiating questions and providing responses differently.

**Discussion:**

This study provides methodological and practical contributions. Methodologically, this study extends prior methods of applying network analysis beyond its original preservice teacher training contexts; practically, this study provides strategies to practitioners to support students’ asynchronous forum discussions.

## Introduction

Systems thinking, a reasoning process within and between levels of biological organization and across system components, is a higher-order thinking skill important for medical students (Lira and Gardner, 2017). It is hard to grasp partially because it requires students to organize system components and processes in an interconnected framework and understand the underlying law that governs the emergence and the decentralized interactions of a system (Michael, 2007; Assaraf and Orion, 2010). In addition, it is challenging to support students’ systems thinking development. Traditional instructor-led lectures constrain students’ systems thinking development as they fail to help students reorganize the fragmented conceptual schemata or establish the “components–phenomena” bonding relationships that are considered critical for developing systems thinking (Hmelo et al., 2000; Hmelo-Silver et al., 2007; Michael, 2007; Liu and Hmelo-Silver, 2009). Researchers designed technology-rich inquiry-based learning environments to support students’ understanding of systems thinking. The underlying rationale is to make invisible components and processes visible and support students’ collaborative inquiry (Hmelo-Silver et al., 2007; 2017). However, these studies were conducted in classrooms and often took several months to implement. Furthermore, as the COVID-19 pandemic continues, it is hard to transform the already worked examples to online settings. Moreover, few studies focused on engaging students in meaningful discourse or investigating what types of discourse features might be more conducive to systems thinking. This makes the transformation from classroom to online settings even harder as students construct understanding mainly through discourse (Scardamalia, 2002). In this study, we explore how to support students’ systems thinking in an online setting during the pandemic. In addition, we also aim to investigate discourse features that are conducive to systems thinking development in online settings to inform educational researchers and practitioners.

Among the tools that support students’ online learning, asynchronous discussion forums have been shown to promote students’ higher-order thinking skills, especially for college students (Chen and Chiu, 2008; Chen et al., 2012). With proper facilitation, students engage in meaningful discourse that are conducive to higher-order thinking skills (Yang, 2019; Yang et al., 2022). The facilitative strategies mainly include prompting questions embedded in the forum such as sentence starters and conversational agents. However, the scripted approach might take away students’ agency and “disenable” students’ capacities to engage in progressive discussions important to systems thinking.

Researchers found that encouraging students to ask questions is an effective approach to enact epistemic agency and engage in progressive discussions and thus, promote higher-order thinking skills (Yang, 2019; Yang et al., 2022). In this study, we aim to promote students’ systems thinking skills, an important higher-order thinking skills for medical students, in asynchronous discussion forums by encouraging students to initiate questions. In the following sections, first, we review the studies that support students’ systems thinking across multiple contexts, including both technology-rich and regular classrooms. We aim to extract feasible design features that support students’ systems thinking. Second, we describe technological affordances of asynchronous discussion forums in supporting students’ systems thinking, the problems of engaging students’ meaningful discourse with prompts and the extent to which encouraging students asking questions support the development of systems thinking.

## Literature review

### Supporting systems thinking in technological and non-technological contexts

Researchers have explored pedagogical strategies to support students’ development of systems thinking. One common approach is to facilitate students’ understanding of how phenomena emerge from interactions between components and how to use mechanisms to explain these phenomena (Hmelo-Silver et al., 2007). In one study, Liu and Hmelo-Silver (2009) examined the effectiveness of organizing multimedia learning materials based on key phenomena-oriented questions (e.g., “How does air get into our body?” and “Why do we need oxygen?”) to promote students’ systems thinking. The results show that students in the experimental group were more likely to mention micro-level processes at the cellular level and make connections between phenomena and mechanisms than their counterparts who learned multimedia materials that were organized around components, such as the lungs or oxygen. Wilensky and Resnick (1999) designed an agent-based modeling tool, Netlogo, to support students’ reasoning about complex systems. The students used the tool to construct understandings of how the properties and behaviors of individual elements interact to create aggregate effects at a higher level. Other researchers have incorporated Netlogo as a constituent of a technological suite to support students in learning about complex systems. For example, Hmelo-Silver et al. (2017) integrated two Netlogo simulations in their technologically rich learning environment to support students’ understanding about the major processes (i.e., photosynthesis, respiration, and decomposition) of an ecosystem. Through this process, the students learned why the death of fish was an emerging phenomenon that was caused by interactions of micro-level (e.g., oxygen) and macro-level (e.g., algae, sunlight, and nutrient run-off) components.

Other researchers have supported students’ development of systems thinking in non-technological contexts. For example, Assaraf and Orion (2010) designed a curriculum suite on understanding the concept of the water cycle to support middle school students’ development of systems thinking. The curriculum was oriented around an overarching question that encouraged the students to explore various components of the water cycle system and how each component interacted to affect the system. Students went on field trips to explore phenomena related to water cycles. Finally, the students engaged in knowledge integration activities and drew models and concept maps to explicate the components and phenomena of the water cycle system. Hmelo et al. (2000) designed a learning environment for a regular classroom that engaged students in “learning by design.” They asked middle school students to design artificial lungs that performed respiratory functions. The students engaged in iterative cycles of designing a prototype, implementing, looking for troubleshooting strategies, asking questions, and collaborative problem-solving. The problems that emerged from the implementation process encouraged the students to connect malfunctioning phenomena with underlying mechanisms and troubleshoot them. Through this process, the students aligned their intuitive understanding with the phenomena emerging from the (mal)functioning of the lungs, asked questions to improve their design prototypes, and solved problems collaboratively.

Although these studies were implemented in both technology-rich and regular classrooms, researchers supported students’ systems thinking with similar strategies. First, they used phenomena questions to engage students thinking at earlier stages of the intervention (e.g., Liu and Hmelo-Silver, 2009; Assaraf and Orion, 2010). Second, in both contexts, students were allowed to identify components, and make connections among these components, explain phenomena emerging from the interactions of the components, and construct understandings of the governing mechanisms. The technological tools afforded the demonstration of components and processes visible to students.

### Promoting students systems thinking in asynchronous discussion forums

Discussion forums were equipped with technological affordances that support students’ development of systems thinking. The forum makes ideas accessible to all learners through displaying ideas in a communal space (Scardamalia, 2002). This is similar with the computational tools that make components salient to students. In addition, the discussion forum affords students to make links of multiple types of information through engaging in meaningful discourse, such as bringing up relevant, important, and novel ideas, referring to inferences, clarifying ambiguities, linking different ideas, making justifications, and widening discussions by asking more questions (Newman, 1995). In addition, students engaged with progressive discussions in the forum, such as responding to the teachers’ prompting questions by elaborating and extending ideas, reflecting upon their learning process, and linking multiple sources of ideas to extend the discussions (Li and Yu, 2020). Hakkarainen and Sintonen (2002) considered such discourse as progressive inquiry in which students engage with iterative cycle of bringing and incorporating new information into the discussion. Such a process is similar with Hmelo et al. (2000) design study in which they engaged students in the iterative process of using malfunction as new information and incorporating such information (phenomena) to progressively understand mechanism.

Students seldom engage in meaningful discourse automatically. Researchers have been designing scaffolds to support students’ meaningful discourse. Alexander et al. (2010) used the “four-questions technique” as discussion prompts to ask students to analyze, reflect, apply, and question educational psychology concepts to promote their online discourse. Studies have also shown that structured prompts, such as explicit instructions on how to generate initial posts and responses, can result in high levels of cognitive and reflective responses (Land et al., 2007; Stegmann et al., 2007; Darabi and Jin, 2013). In addition, Bai (2009) informed a group of students that their discussion posts should include all four phases of cognitive presence (triggering, exploration, integration, and resolution). The results indicated that these students had higher cognitive presence than students who were not informed in this way. Researchers have also used reflective questions to elicit students’ thoughts, such as clarifying ambiguities, probing assumptions, eliciting reasoning and evidence, and encouraging students to reach a consensus (e.g., Yang et al., 2005; Darabi et al., 2011). Wu et al. (2019) investigated the role of adaptive versus static scaffolds in supporting preservice teachers’ design thinking development. The results showed that metacognitive scaffolds, whether static or dynamic, supported students’ design problem-solving process. However, students from the adaptive groups tended to be more focused on holistic and interdisciplinary learning design solutions than their counterparts.

In most of the studies, the teacher assigns several discussion topics to students using technological tools or pedagogical strategies as scaffolds which are conducive to meaningful discourse. However, few studies have examined the effectiveness of teachers encouraging students to initiate questions and explore these questions. Encouraging students to ask questions is critical (Hakkarainen, 2003), partly because questions drive scientific inquiry and asking questions are indicators of students’ epistemic agency (Stroupe, 2014). Prior studies also showed engaging students asking questions would lead to meaningful discourse (Scardamalia, 2002). In addition, phenomena-oriented questions enhanced students’ systems thinking (Liu and Hmelo-Silver, 2009). In this study, we examine whether engaging students with meaningful discourse *via* students initiating questions and participating in online asynchronous discussions promote systems thinking. We address the following research questions:

1. How do students perceive the effectiveness of initiating questions in discussion forums in supporting systems thinking development?
2. To what extent do students’ question initiation and online discussion participation promote systems thinking development?
3. How do students with different participatory roles engage in meaningful discourse differently?

a) Do students with different participatory roles ask systems thinking questions at different levels and provide different levels of responses?
b) What characterizes students’ meaningful discourse with different participatory roles?

## Materials and methods

### Instructional context

This study took place in a 16-week mandatory course in a medical school in China. The participants included 22 junior students, all of whom were women. The course was about midwifery and related concepts, such as pregnancy, delivery, and postpartum care. The course was taught by four instructors, each of whom led a few units. After the outbreak of the pandemic, the course went fully online. Students were then asked to watch video lectures, attend synchronous video conferences, review course reading materials, participate in asynchronous discussions, and take online assessments. All of the students had stable internet access throughout the course.

### Study design

One of the co-authors provided 4 weeks of the instruction in this course. During these weeks, two units were covered: one on nursing care for women with complications during pregnancy (unit 1, weeks 2–3) and the other on home care for newborns (unit 2, weeks 5–6). To compare the students’ learning between two different pedagogical approaches, the instructor designed Unit 1 using traditional instructor-led learning activities, in which the students watched video lectures and attended synchronous video conferences to mimic face-to-face teaching. Unit 2 incorporated an inquiry-based pedagogical approach, in which the students were asked to read the assigned readings, ask questions in discussion forums, and participate in student-led discussions. The instructor provided timely interventions on the forum, which included direct instruction and meta-cognitive prompts, such as “*x*, why do you think *y* posed a good question?”

### Data collection

The data sources are described in Table 1. The instructor posted three open-ended questions for the students to answer individually by the end of each unit. These questions asked the students to explain why certain phenomena happened and aimed to measure the students’ systems thinking levels. Correct answers could not be simply located on any learning materials. The questions were reviewed by three medical education experts to examine their validity for measuring students’ systems thinking levels. The questions for each unit are listed in Table 2. In addition, as part of the inquiry-based pedagogical approach, the students posted questions and made replies in the forum. These questions covered the key concepts in Unit 2, such as home care for newborns. The teacher organized the questions into different sections according to the key concepts to which they were related.

**Table 1:** Data sources for units 1 and 2.

Data source	Unit 1	Unit 2
Open-ended questions	✓	✓
Student-generated questions		✓

**Table 2:** Open-ended questions.

No.	Unit 1 question	Unit 2 question
1.	If the patient has palpitations or labored respiration, which system should be considered as having a problem? (What has caused the symptoms?)	Before the perineum rinsing operation, how do you conduct the evaluation? Which are the key points to be evaluated?
2.	If the pregnant woman is experiencing palpitation or labored respiration, what might happen to the fetus?	If lateral peritectomy and suture are to be performed on the pregnant woman, which items should be further evaluated before the perineum rinsing operation?
3.	How do you provide health guidance to a pregnant woman with GDM at 26 weeks of gestation? (Which aspects should be considered?)	Case study: A newborn 3 days after birth cries during a bath. How do you handle this situation?

### Data analysis

**Figure 1:**
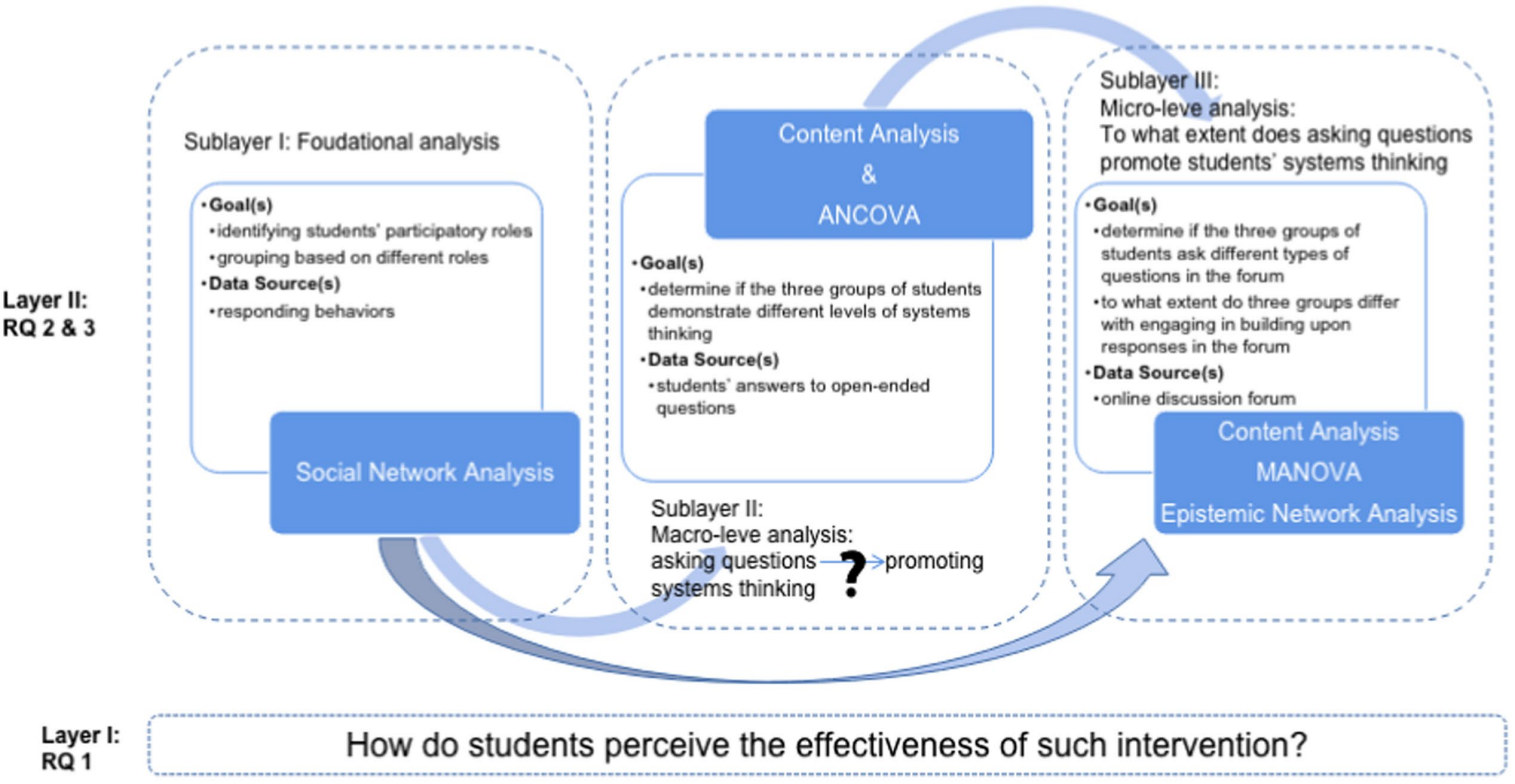
Analytical framework.

We conducted two layers of analysis to investigate whether and how encouraging students to ask questions in discussion forums supports systems thinking development (see Figure 1). In the first layer, we conducted an overall investigation of the effectiveness of the learning environment in promoting students’ systems thinking skills, with data collected from interviews. The second layer was constituted by three sublayers. In Sublayer I, we used centrality metrics computed from a social network analysis (SNA) to identify the different participatory roles of the students. This sets the foundation for further analyses. In Sublayers II and III, we used content analysis, a set of inferential statistical analyses, and an epistemic network analysis (ENA) to examine whether students with different participatory roles demonstrate different levels of systems thinking and how they engage differently in online discussion.

#### Layer I

Semi-structured interviews were used to probe the students’ perceptions of the intervention. We asked the students three open-ended questions: (1) Do you think this is an effective approach to promote your understanding of key concepts? (2) Why do you think this is an effective approach to promote your understanding? (3) Will you use this approach spontaneously in your future study? All interviews were conducted individually using the Zoom teleconferencing software with the researcher and student present and the classroom teacher absent. Each interview lasted for 20–30 min. The interviews were audio recorded.

The interview data were transcribed, imported, and coded in NVivo 12. We used a constant comparative method to identify themes in the interview data (Charmaz, 2014). Specifically, we conducted a two-phase analysis. In Phase 1, we constructed a holistic understanding of the interview transcripts. We identified and defined eight different codes to students’ explanations of why the approach was effective. In Phase 2, we used the constant comparison approach to collapse those themes identified in Phase 1 that were narrowly defined but related in meaning. From this procedure we arrived at two major themes.

#### Layer II, Sublayer I

Three types of centrality metrics were used to characterize the students’ participation. The degree centrality of a network represents the number of ties directly connecting to one node; the more direct connections there are, the higher the degree centrality is. In a directed graph, the degree centrality includes in-degree, representing how many nodes are connected with one node, and out-degree, representing how many connections go out from one node. The closeness centrality is the sum of the shortest paths from all other nodes to one node, and the betweenness centrality reflects to what extent one node is located on the shortest path connecting other nodes. The value of a node’s betweenness centrality indicates the power of that node in controlling information flow in a network.

As we were interested in the breadth rather than the depth of students’ connections with peers, we transformed the students’ responding behaviors to a directed, unweighted interaction dataset. We used UCINET (Version 6) to compute the normalized in-degree, out-degree, closeness, and betweenness centrality. We used harmonic closeness centrality to represent students’ closeness centrality, as there were multiple unconnected nodes in the network. We ranked the centrality scores and assigned different levels to each centrality metric: specifically, centrality scores ranked among the top 30% of all individual students’ centrality scores were considered “high,” scores ranked in the top 70% but not in the top 30% were considered “medium,” and scores not in the top 70% were considered “low.” We further identified the students’ participatory roles by reference to their rankings. Students were designated as leaders if either their degree or closeness centrality ranking was high and their betweenness centrality was high; as peripheral participants if either their degree or closeness centrality was low and their betweenness centrality was zero (reflecting students who were in a peripheral position in the network and seldom made connections with peers); and as regular participants if they did not meet the criteria for either the leader or peripheral positions, with degree and closeness centrality at medium levels.

**Figure 2:**
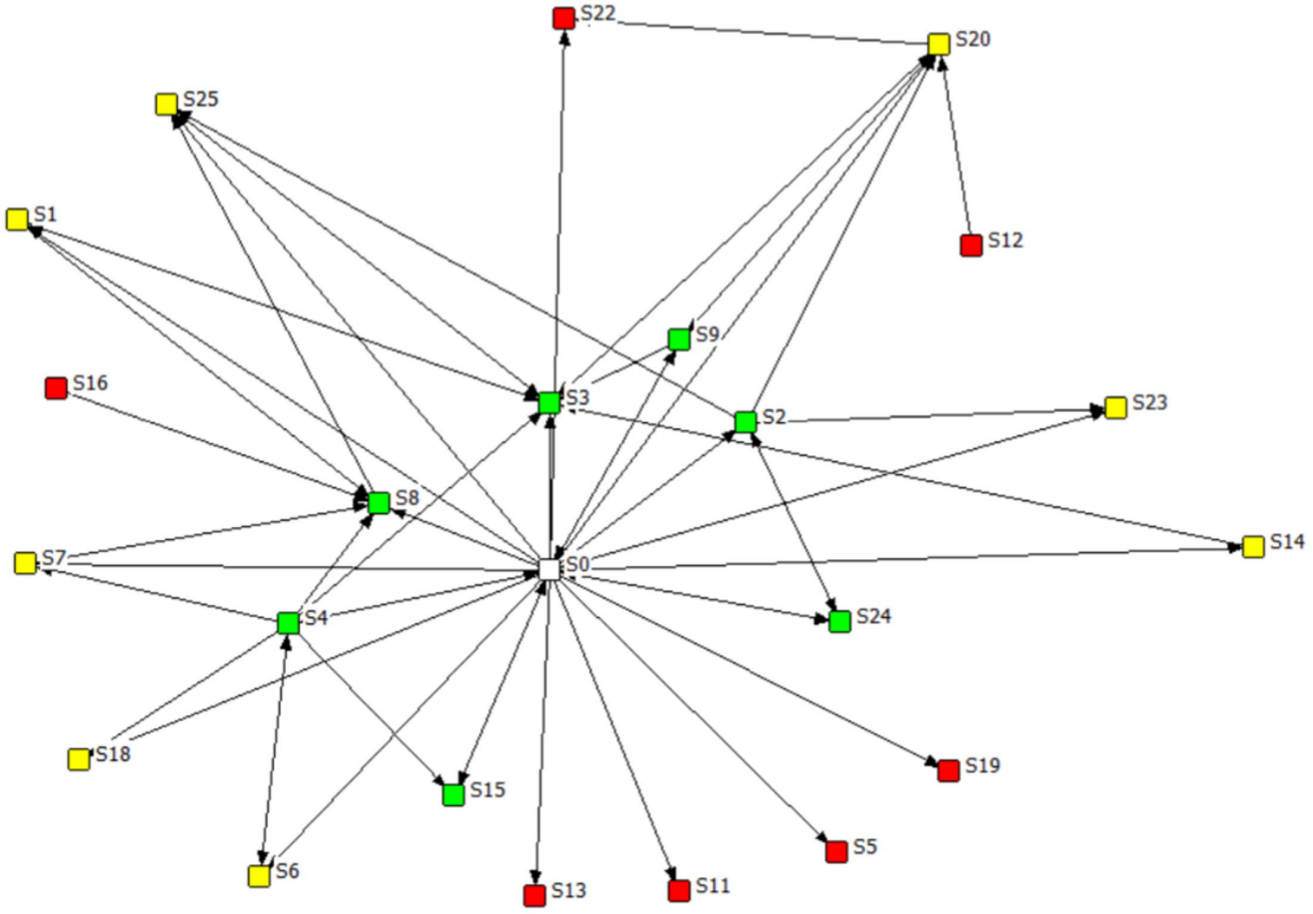
Students’ participation network. The green squares are participants identified as leaders, the yellow as regular, and the red as peripheral.

Teachers play an important role in facilitating online discussions. We included a teacher node in the SNA and explored how the students participated against the backdrop of the teacher’s active facilitation. As the teacher had high centrality values, we placed her as the central node of the participation network (see Figure 2).

#### Layer II, Sublayer II

The content analysis in Sublayers II and III was conducted in three phases. In Phase 1, we reviewed the relevant literature. In Sublayer II, we categorized students’ systems thinking into four levels according to prior studies (Hmelo-Silver et al., 2007; Eberbach et al., 2012). Hmelo-Silver et al. (2007) identified middle school students’ trajectory of understanding systems thinking from identifying structures, progressing to connecting structures and behaviors (or functions) and ultimately using mechanisms to explain the behaviors and functions. In addition, Eberbach et al. (2012) identified a three-level trajectory characterizing middle-schoolers’ thinking progress, starting from recognizing one cluster of phenomena, moving to identifying multiple un-connected clusters, and finally arriving at coherent and meaningful connections among the clusters. In this study, we identified first level as students’ egocentric perspective and believing everything happening in the system is detrimental to the human body, second level as using some phenomena to explain other phenomena without unpacking any mechanisms, third level of identifying a certain mechanism of the phenomenon, but only making connections on one level of the phenomenon rather than across multiple levels and fourth as locating multiple mechanisms of the phenomenon and connecting them with appropriate relationships (see Table 3). In Sublayer III, we coded the questions with van Aalst’s characterization of different question types and coded the responses with Newman (1995) conceptualization of how students engage with social critical thinking processes in online learning modes (see Table 4 for the coding scheme). In Phase 2, we applied the two coding schemes to one third of the data and convened to discuss the discrepancies and unit of analysis, collapse similar codes, and settle our coding schemes. Our analysis unit for Sublayer II was an individual student’s answers to the open-ended questions and for Sublayer III was one note (either a question or a response note). In Phase III, we coded the remainder of the data. The inter-rater reliability was high: for explanation-seeking questions, the inter-rater reliability was 97.5%; for fact-seeking questions, it was 98.05%; for responses, R.3.4 = 87.65%, R.4.2 = 81.62%, R.6.1 = 87.67%, and R.8.1 = 89.13%; and for sustaining inquiry, it was 91.62%. This analysis formed the basis of the subsequent statistical analysis and ENA.

**Table 3:** Coding scheme for scoring student answers in systems thinking.

Code	Description
1	Egocentric perspective and believing everything happening in the system is detrimental to the human body
2	Using some phenomena to explain other phenomena without unpacking any mechanism
3	Identifying a certain mechanism of the phenomenon, but only making connections on one level of the phenomenon rather than across multiple levels
4	Locating multiple mechanisms of the phenomenon and connecting them with appropriate relationships

**Table 4:** Coding schemes for students’ question and response types.

Code (ENA abbr.)	Definition
Fact-seeking question (IQ. Facts. Questions)	Question that asks for facts or information
Explanation-seeking question (IQ. Explanation. Questions)	Question that asks for an explanation, or open-ended questions
Referring to external material (R.3.4)	Response that refers to resources other than the assigned learning material and textbooks
Referring to course material (R.4.2)	Response that refers to the assigned learning material and textbooks
Linking ideas, facts, and notions (R.6.1)	Drawing link between ideas and factual information from peers to construct a new understanding
Critical assessment of own/others contribution (R.8.1)	Assessing to what extent their ideas or those of others aid in their understanding
Sustaining inquiry question (sustaininquiry)	Asking further questions based on prior students’ responses

ENA, epistemic network analysis.

With the content analysis of the students’ answers to the open-ended questions, we assigned individual students one point for every open-ended question in each unit and summed the points to generate a total systems thinking score for each unit. We conducted a one-way analysis of covariance (ANCOVA) to examine whether the three groups of students demonstrated different levels of systems thinking. We used the bootstrapping strategy in SPSS to compensate for the small sample. The covariate was the systems thinking score in Unit 1, the independent variable was participation level, and the dependent variable was the systems thinking score in Unit 2.

#### Layer II, Sublayer III

We used the same grading criteria as that used in the open-ended questions to assign separate scores to students’ questions and responses. A one-way multivariate analysis of variance (MANOVA) was conducted to examine whether the three groups of students scored differently on systems thinking scores in questions and responses. We again adopted the bootstrapping strategy. The two types of scores were the dependent variables, with the three different participatory roles as a fixed factor with three levels: leader, regular, and peripheral.

For the content analysis of the students’ online posts, we conducted an ENA, which is used to model and visualize the co-occurrence of codes. An ENA can describe the interrelationships among different dimensions of codes in a student’s cognitive framework and reveal students’ learning process by demonstrating how different conversational codes occur and interact and how students’ cognitive skills develop over time (Shaffer et al., 2009). ENA has been applied to the analysis of students’ textual data to examine the effectiveness of an intervention by comparing students’ knowledge construction at various stages and under different conditions. The analytic tool assists researchers in comparing the skill development trajectory exhibited by students as an intervention unfolds and how different intervention conditions support the development of students’ cognitive skills (Wu et al., 2019; Sun et al., 2022). Our aim in this study was to examine how students from different participatory roles engage differently in online discussions: specifically, the different types of questions they asked and the different forms of engagement they demonstrated in building upon each other’s responses. We used the students’ grouping results as the unit of analysis. The stanza was a single discussion thread, comprising one question and several responses. The sliding window size was an entire conversation, which is a complete discussion thread.

## Findings

### Students’ perception of the effectiveness of initiating questions in discussion forums in supporting systems thinking development

Nineteen of the 22 students were interviewed. All the interviewees thought that the approach was effective in supporting systems thinking development. On the reasons for students perceiving the intervention as effective, the following themes emerged.

First, asking questions encouraged students to make connections among different sources of information, including their preexisting knowledge, intuitive understandings, and perceptions from daily life. Several students mentioned that they were motivated to ask questions by conflicts between their intuitive understanding and the authoritative information provided on the video or other learning materials. For example, one student mentioned, “The difference between the practical experience from my internship and the content in the learning material prompts me to ask questions.” Other students mentioned that they were connecting prior knowledge with new information from the learning material. For example, one student stated, “In the part on neonatal touch, there are many acupoints in our human body, so why stop there and why not find out which acupoints it will stimulate? I had learned in other courses that the human body has many acupoints. [In asking the question] I was just connecting the knowledge of this course with my previous knowledge.”

Second, the students asked questions because they wanted to know the underlying rationale for certain procedures. Some students mentioned that they typically referred to learning resources to understand the sequence of certain procedures, or why a given procedure should be followed by another. Others said that they asked questions after connecting their laboratory experiences with the theory taught in the course. For example, one student mentioned, “When it’s a theory class, I need to understand why a symptom happens and how to deal with it—I need to know how to complete the operation smoothly and also understand the purpose of each step; but I am also relating the two.”

In summary, almost all students who perceived the effectiveness of this intervention suggested that asking questions was a good way for them to make connections between phenomena and underlying mechanisms which has been identified as an important characteristic of improved systems thinking.

### Identifying and characterizing students’ participatory roles

Among the 22 students who participated in the study, seven were identified as leaders, eight as regular participants, and seven as peripheral participants. The means and standard deviations of the students’ centrality scores on each dimension are reported in Table 5.

**Table 5:** Means and standard deviations for centrality scores by participation group.

Group	*n*	Out-degree		Out-closeness		In-degree		In-closeness		Betweenness	
		M	SD	M	SD	M	SD	M	SD	M	SD
Leader	7	0.06	0.03	0.33	0.11	0.04	0.03	0.39	0.22	2.44	2.30
Regular	8	0.03	0.02	0.29	0.06	0.02	0.01	0.26	0.21	1.09	1.71
Peripheral	7	0.01	0.02	0.18	0.12	0.00	0.01	0.06	0.12	0	0

Figure 2 illustrates the students’ participation network. The green dots are the students identified as leaders, the yellow dots are regular, and the red dots are as peripheral. As the teacher actively facilitated the students’ discussions and replied in every thread, the core of the network (represented by the white dot) is the teacher, who had the highest centrality score. The students in the leader group are located around the center of the network. They were either actively responding to peers or being responded to by other peers. For example, multiple students responded to S3, S8, and S2. They also made connections within their own group. For example, S2 and S24 made mutual connections. In addition, compared to the students in the other groups, those in the leader group served more as bridging nodes in the network, connecting students from other groups. This is consistent with the high betweenness centrality of the leader group, as shown in Table 5. In contrast, the students in the peripheral group had connections only with the teacher, suggesting that these students’ questions were only responded by the teacher. Furthermore, only two of the peripheral students (S12 and S16) responded to a question. This indicates that the peripheral students made few connections with their peers. The students in the regular group showed a medium level of participation. Although they made connections between the students in the leader group, they made no connections within their own group, which demonstrates the looseness of their connections in the network.

### To what extent does participating in online discussions promote students’ systems thinking skills?

The means and standard deviations for each of the three groups of students are shown in Table 6. Before running the ANCOVA, we checked for any violation of homogeneity of variance. The result showed that there was no interaction between the covariate and independent variables, *F* = 0.57, *p* > 0.05, indicating that an ANCOVA was appropriate to interpret the difference in systems thinking level across the three groups. Table 6 shows that there were significant differences between the three groups on students’ systems thinking scores in Unit 2.

**Table 6:** Means and standard deviations for unit 2 systems thinking scores by participation group.

Group	*n*	Adj. M	*SD*	*F*
Leader	7	7.83	0.41	20.66***
Regular	8	5.25	0.39	
Peripheral	7	4.02	0.43	

***p < 0.001.

To further investigate whether the students in the leader group demonstrated higher levels of systems thinking than those in the other groups, we conducted a set of pairwise comparisons. Using the least significant difference approach, the adjusted mean systems thinking score in Unit 2 for the leader group was significantly different from that of the regular (*p* < 0.001) and peripheral (*p* < 0.001) groups, but there was no significant difference between the regular and peripheral group (*p* = 0.055). Controlling for their baseline systems thinking abilities in Unit 1, the students in the leader group outperformed those in the other two groups. This indicates that more active participation in online discussion was associated with higher levels of systems thinking in the post–learning unit assessment.

### Characterizing students’ participation in the three groups

#### Statistical analysis of the students’ participation in the three groups

In total, students posted 79 messages in the discussion forum. Leader students posted 45 notes, among which 12 were explanation questions, 4 facts-seeking questions and 6 sustaining inquiry questions. They initiated 70.6% of all explanation questions, 22.2% of all facts-seeking questions and 100% of all sustaining inquiry questions. In addition, they provided 23 responses which account for 60.5% of all responses. Regular students posted 26 notes, among which 5 were explanation questions, 9 facts-seeking questions and 0 sustaining inquiry questions. They initiated 29.4% of all explanation questions, 50.0% of all facts-seeking questions. In addition, they provided 12 responses which account for 31.6% of all responses. Peripheral students posted 8 notes, among which 5 were facts-seeking questions, 3 responses. Detailed figures are shown in Table 7.

**Table 7:** Discussion forum question and response types by participation group.

Group	Initiating questions		Responses	
	Explanation-seeking (%)	Fact-seeking (%)	Sustaining inquiry (%)	Others (%)
Leader	12 (70.6%)	4 (22.2%)	6 (100%)	23 (60.5%)
Regular	5 (29.4%)	9 (50.0%)	0 (0%)	12 (31.6%)
Peripheral	0	5 (27.8%)	0 (0%)	3 (7.9%)
Total	17 (100%)	18 (100%)	6 (100%)	38 (100%)

The means and standard deviations of the students’ systems thinking scores across the three groups are presented in Table 8. Box’s *M* was not significant (*M* = 5.641, *p* = 0.196), indicating that there were no significant differences between the covariance matrices. Thus, a MANOVA could be applied. The result of MANOVA suggested there are significant difference across three groups in both systems thinking scores (Wilks’ Lambda = 0.136, *F* = 15.435, *p* = 0.000, partial *η^2^* = 0.632). Follow-up univariate tests indicated significant differences on systems thinking levels in initiating questions (*F*(2, 19) = 38.052, *p* = 0.000, partial *η^2^* = 0.8) and responses (*F*(2, 19) = 12.072, *p* = 0.000, partial *η^2^* = 0.560). The results indicated that the three groups of students asked different levels of systems thinking questions and provided different levels of responses. In particular, the students in the leader group contributed questions and responses that involved significantly higher levels of systems thinking.

**Table 8:** Means and standard deviations of systems thinking scores by participation group and post type.

Group	*n*	Post type	*M*	SD
Leader	7	Questions	6.29	1.89
		Responses	5.71	3.15
Regular	8	Questions	2.38	0.92
		Responses	2.38	1.77
Peripheral	7	Questions	0.71	0.49
		Responses	0.29	0.49

#### Characterizing the students’ participation patterns with different participatory roles

**Figure 3:**
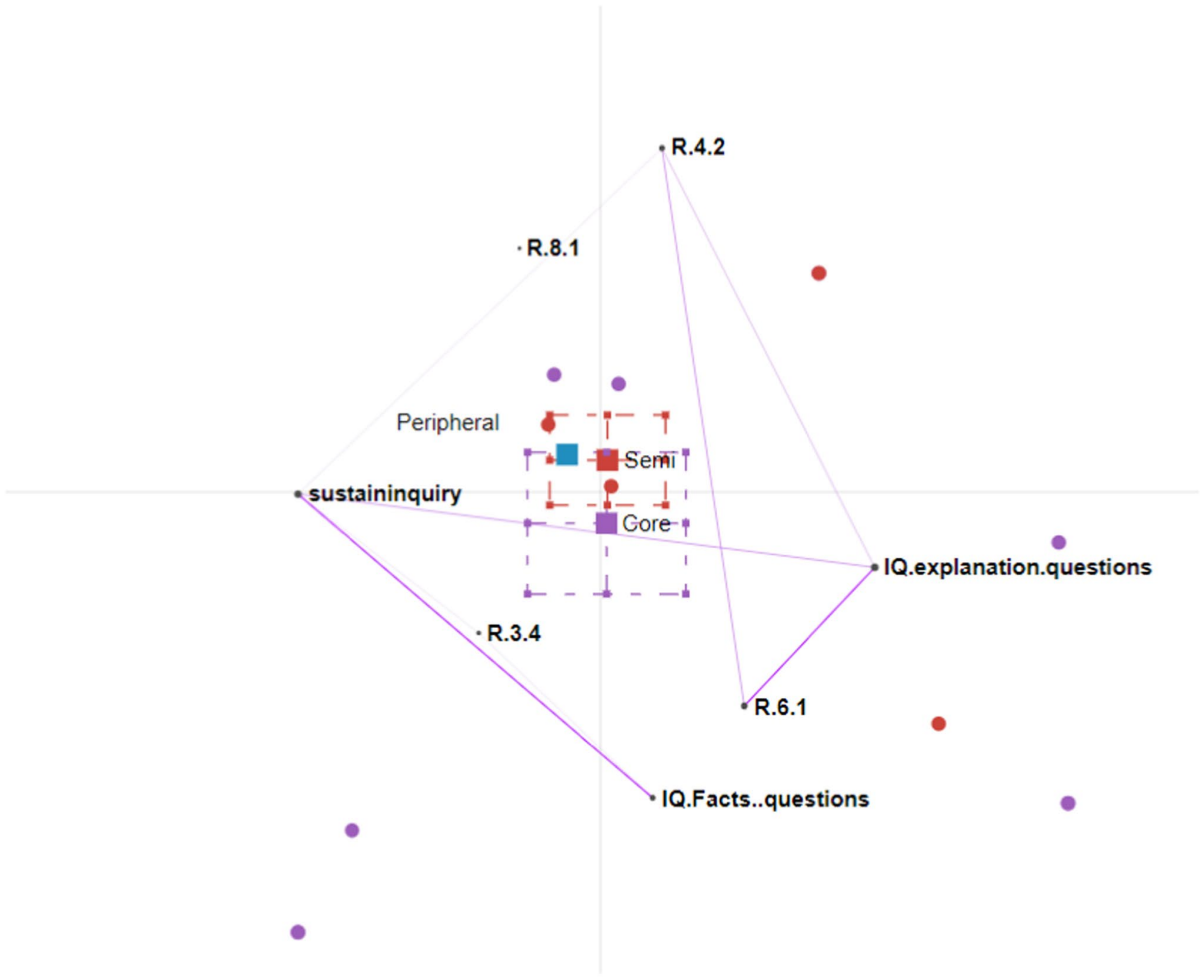
Epistemic network analysis participation graph for leader group. Core = leader; semi = regular.

**Figure 4:**
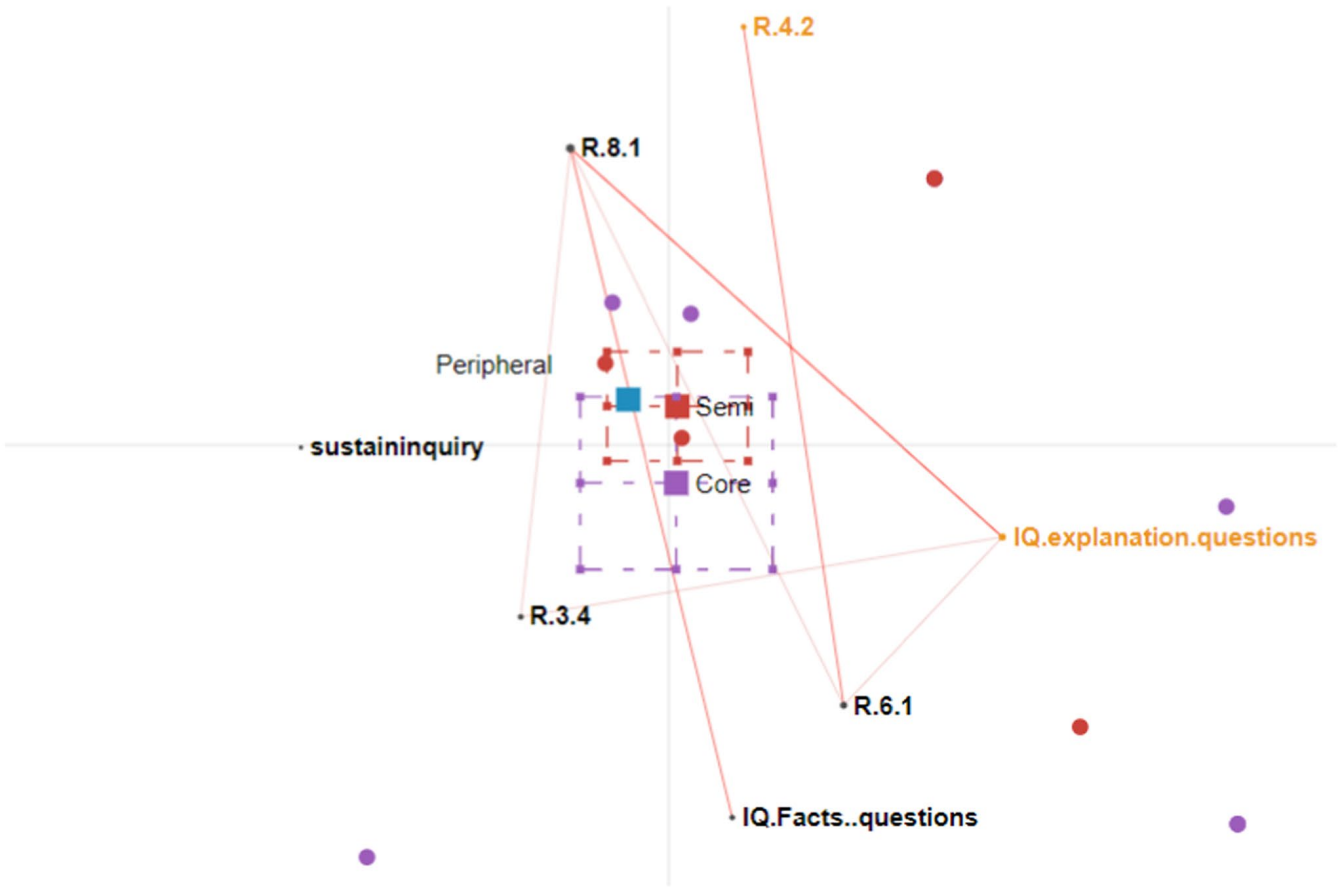
Epistemic network analysis participation graph for regular group. Core = leader; semi = regular.

**Figure 5:**
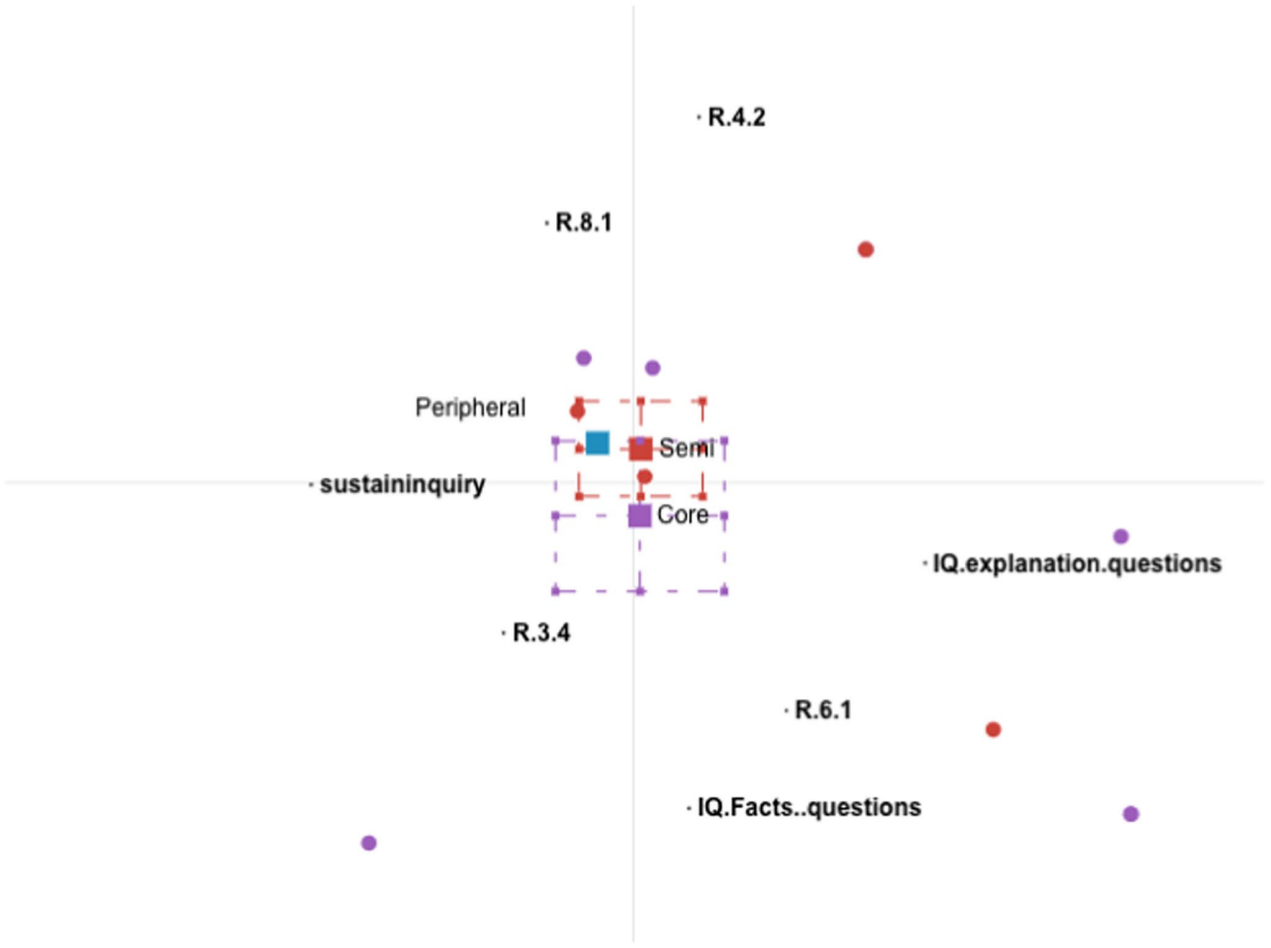
Epistemic network analysis participation graph for peripheral group. Core = leader; semi = regular.

**Figure 6:**
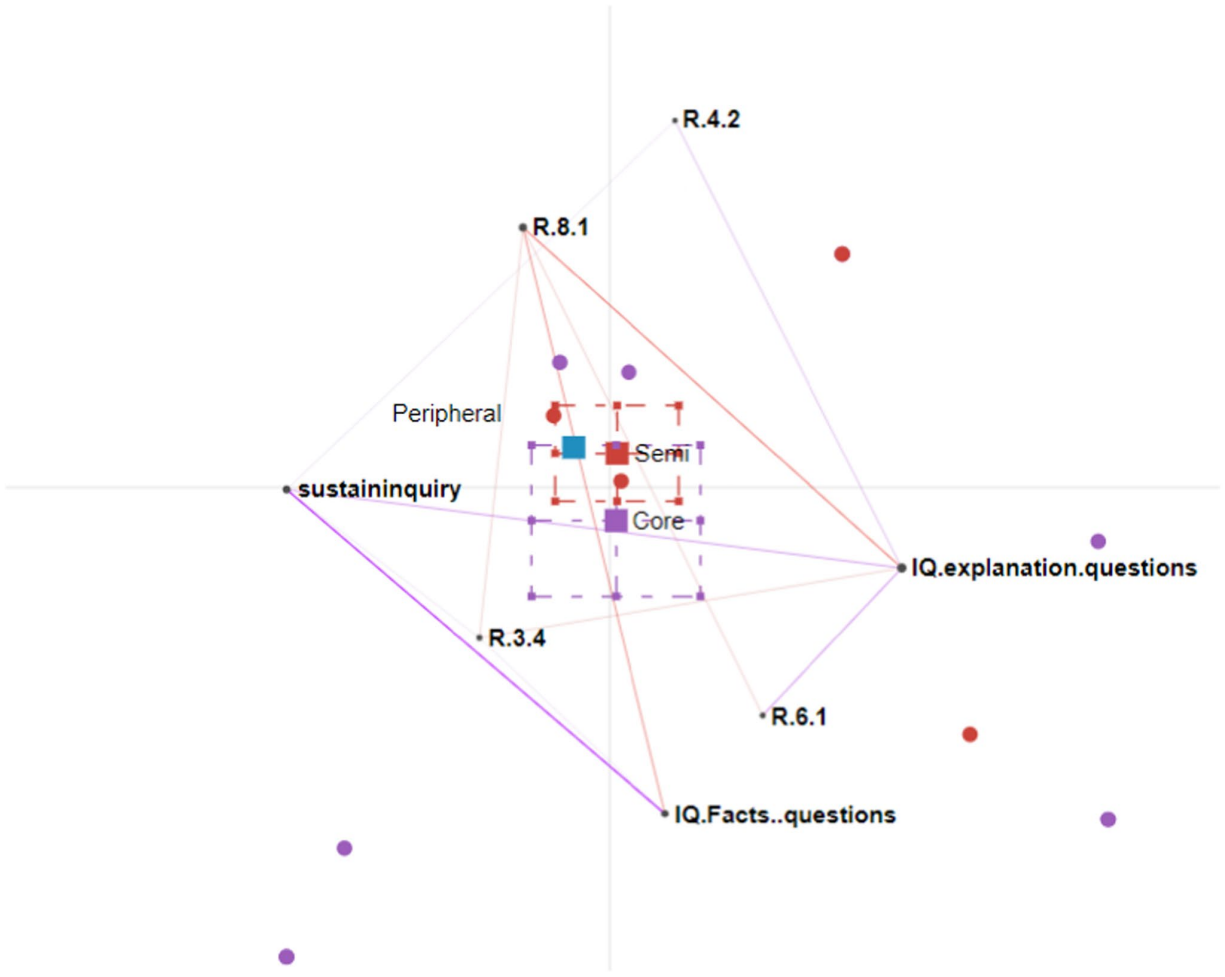
Epistemic network analysis graph comparing the regular and leader groups. The leader group’s connections are shown as purple lines; the regular group’s connections are shown as red lines.

We conducted four rounds of ENA analysis. Figures 3–5 show the participation patterns of students in the leader, regular and peripheral group, respectively, and Figure 6 shows comparison of key features between leader and regular groups.

##### Participation characteristics of the students in the leader group

The students in the leader participation group continuously improved their understanding by making links between diverse information and ideas. They incorporated new information and asked further questions to sustain the inquiry in discussion threads.

The students in the leader group asked explanation-seeking questions, which encouraged them to refer to course material to provide explanations, to incorporate different sources of information, and to link ideas, facts, and notions. This is demonstrated by the triangle on the ENA graph connecting explanation questions, R.4.2, and R.6.1. For example, one student asked, “Under what kind of situation can one not perform a breast massage?” Students responded with multiple explanations referring to the course materials, such as “Women with suppurative mastitis, breast tumor, and other diseases should not have a breast massage” and “Those with local redness or induration at the nipples or chapped breasts cannot receive a breast massage.” Building upon these explanations, one student responded, “Patients with acute mastitis and breast cancer should not have a breast massage because it will promote the spread of inflammation or cancer cells.” In this example, a student asked an explanation-seeking question and elicited explanations from multiple perspectives. Another student then summarized these explanations and linked the ideas about breast massage with the spread of cancer cells. This demonstrates high-level systems thinking, as she made connections between symptoms (no breast massage) and underlying mechanisms (promoting the spread of inflammation or cancer cells).

As new information was continuously brought up by the students, they asked new questions to sustain the inquiry. This is demonstrated from a line connecting “refer to course material” (R.4.2) and “sustaininquiry.” For example, one student asked an explanation-seeking question, “Why do we need to suddenly stop behind newborns’ ears when touching their head”? Another student explained, “At that time, it should be gently pressed on the mastoid process behind the ear.” A third student then asked a further question: “Why do you want to press it gently on the mastoid?” In this example, the student took up the new information brought by her peer and asked a further question to extend her understanding.

Both explanation-seeking questions and fact-seeking questions encouraged the students to ask further questions to sustain the inquiry. This is demonstrated on the ENA graph by the lines connecting explanation-seeking questions, fact-seeking questions, and sustaining the inquiry. The students asked further questions when they were either unsatisfied with the answers provided by the teacher or willing to extend the inquiry by introducing a new perspective. For example, one student asked the following question: “Negative pressure suction is often used to correct sunken or flat nipples, but this method can cause uterine contractions and may cause premature birth and miscarriage. Why this method is still being used?” The teacher answered, “At present, the method of pulling the nipple can be used to correct nipple depression or flat nipple during pregnancy, so as not to cause uterine contraction.” The student (S4) who had asked the initial question returned to the conversation and asked a further question: “Is there any way to correct it other than negative pressure suction?” In this example, the student had asked an explanation-seeking question that challenged the validity of an operation and the teacher had provided an explanation, but it seems that the student was not satisfied and thus asked a further question. The student evaluated the teacher’s answer, which could be considered as new information, and asked a further question.

##### Participation characteristics of students in the regular group

Students in the regular group asked both explanation-seeking and fact-seeking questions. The explanation-seeking questions elicited students bringing diverse ideas to the discussion thread and critically assessing the ideas of others. This is shown by the lines connecting explanation-seeking questions, R.3.4, and R.8.1 on the ENA graph. For example, one student asked the explanation-seeking question, “Why should breast milk be applied around the nipple after breastfeeding? Will this increase the risk of infection?” Another student explained, “Breast milk is painted around the nipple to protect the nipple and prevent chapping.” A third student then challenged this explanation by returning to the idea of infection: “I am thinking it might get infected if I put breast milk around.” In this conversation thread, one student provided an answer to the question by bringing new information to the discussion, which inspired another student to evaluate and assess her ideas.

The students continuously connected facts, ideas, and notions from different sources, which inspired critical evaluations and assessments by other students. For example, one student asked the explanation-seeking question, “How do we distinguish mastitis from lactation fever?” The students responded with diverse ideas and made connections among these ideas. For example, one student answered as follows: “Lactation fever occurs when the postpartum mother begins to secrete milk, and the body temperature gradually rises to more than 38 degrees. After dredging the breast duct in time, it can drop by itself. Mastitis is characterized by redness, swelling, thickening, and even ulceration.” Other students also made connections between different symptoms. For example, another student answered, “Mastitis will have systemic symptoms, chills, high fever, and accelerated pulse.” Another student was inspired to evaluate these diverse explanations and responded as follows: “These are all surface features. I just checked the learning material, and mastitis should have increased white blood cells whereas lactation fever generally does not.” In this conversation, the students proposed diverse explanations that inspired others to check the learning materials and evaluate the extent to which the information answered the original question.

##### Participation characteristics of students in the peripheral group

Shown in Figure 6, no lines were observed among codes. This suggests no two codes appear simultaneously in one discussion topic. According to the descriptive statistics of students’ participation, all of their facts-seeking questions were only responded by the teacher, and they responded to peers’ questions with fragmentary information referred from learning materials.

##### Comparison plot of the leader and regular groups

The comparison graph in Figure 6 shows the extent to which the participation patterns in the leader group differed from those in the regular group. There are multiple lines on the graph connecting sustaining the inquiry with other nodes, such as explanation-seeking questions, fact-seeking questions, and nodes representing the students’ responding patterns. This indicates that the students continuously incorporated new information, evaluated this new information, made connections, and asked further questions to sustain the inquiry. However, no connections were observed in the regular group between sustaining the inquiry and other nodes. The students in the regular group critically evaluated their peers’ responses and declared their agreement or disagreement without elaborating on the reasons. However, this usually brought the conversation to an end.

In summary, students in the leader group continuously made connections between different sources of information. They incorporated new information, critically evaluated this new information, and extended discussions by asking further questions. In contrast, the students in the regular group incorporated new information from learning materials or other resources but judged this information without extending the inquiry, thus bringing the conversation to an end.

## Discussion

### General discussions of research findings

In this study, the results showed that engaging students in asking questions in an asynchronous discussion forum supports their systems thinking development, especially for students who actively participate in the discussion. We identified a trajectory that characterized how different participation levels associated with different levels of discourse engagement and how would this affect students’ systems thinking development. Our findings extend existing studies that focus on supporting systems thinking development with technological and computational tools to supporting meaningful discourse and specifically, identifying characteristics of meaningful discourse that are conducive to systems thinking.

Our finding suggests engaging in meaningful discourse promotes systems thinking development. The more active students’ participation was, the better systems thinking outcomes were observed in the post-unit assessment. The qualitative finding withdrawn from ENA further showed that leader students engaged with discourse moves that are not only conducive to high-order thinking skills but also systems thinking. For example, both in regular and leader groups, students continuously brought in new information, made connections among multiple sources of information, and evaluated the validity of such information. This is partly because the discourse moves engage students with similar reasoning patterns as the ones supported by the technological tools. Technological tools support systems thinking by encouraging students to make connections among components with underlying mechanisms to explain emerging phenomena (Wilensky and Resnick, 1999; Hmelo-Silver et al., 2007; 2017). In our study, students engaged with discourse moves that are initiated by questions probing explanation of phenomena. Such type of question elicited discourse moves such as making connections among different components, evaluating the validity of explanations, and asking new questions that probe explanations revealing mechanisms.

Our study supported students’ meaningful discourse by encouraging students to ask questions. The results suggested this pedagogical strategy engaged most students in meaningful discourse which leads to systems thinking development. We consider this might be because students have opportunities to engage with dual rounds connections making. The interview results showed that while students were deliberating questions, they were comparing their understandings with authoritative information, considering how to deal with the anomalies between their existing understanding and new information, and articulating the anomalies as questions. This is what Stahl (2000) mentioned as personal knowledge construction. When students were posting questions in the forum and answering the questions, they were engaging in the social knowledge construction process which was characterized as attending to peer discussions, making connections with personal cognitive schemata, exploration of external authoritative information to explain anomalies, developing alternatives, sustaining of inquiry, and integration of knowledge from various resources. Moreover, the affordance of an asynchronous discussion forum gives students a venue to ask questions and engage in discussions formulating new connections among diverse questions and responses. It makes ideas accessible to all students (Scardamalia, 2002). The teacher acted as a facilitator throughout the discussion by providing direct answers and epistemic guidance. We consider this demonstrates similar pedagogical affordances to teacher presence in CoI (Garrison et al., 2001). Garrison highlighted two essential benefits of a strong teaching presence in student discussion forums: strengthening the sense of a learning community and encouraging students to engage in further inquiry. In addition, the cognitive and epistemic scaffolds support students to engage in progressive inquiries that involve an iterative process of questions and new information integration (Hakkarainen and Sintonen, 2002; Hakkarainen, 2003).

Our ENA result showed how students from different participation levels engaged in meaningful discourse differently and how would these norms affect their systems thinking development. Students in the peripheral group asked facts-seeking questions and provided fragmentary, facts-oriented responses to questions posed by peers. They never linked multiple types of information and advanced collective understanding. Few connections suggest low systems thinking. The leader group asked explanation-seeking questions, linked diverse information and ideas, continuously incorporated new information, and asked further questions to sustain the inquiry discussion thread. They made extensive and intensive connections among diverse pieces of information, thus explaining why systems thinking was promoted most strongly in the leader students. The regular group demonstrated similar participation patterns, but the qualitative analysis showed that they tended to prematurely terminate discussions by judging their peers’ ideas. This inhibited them from making deep or broad connections. Students’ participation norms in the three groups showed a similar trajectory as van Aalst’s knowledge sharing, construction, and creation discourse (Aalst, 2009). In van Aalst’s study, students’ deep learning was promoted as they were engaging with knowledge construction and creative discourse. In our findings, students’ systems thinking was promoted most in the leader group who continuously linked multiple types of information and sustained the inquiry with extended questions.

Furthermore, by comparing different discourse features in leader and regular groups, we found two key discourse types that are essential for systems thinking development. First, explanation-seeking questions elicited responses that referred to authoritative information, linked different strands of ideas, and inspired further questions emerging that sustained the inquiry. In contrast, fact-seeking questions elicited responses expressing judgments or evaluations. These results echo those of the science education literature, which has found that questions drive inquiry. In knowledge-building studies, Tong and Chan (2019) demonstrated that explanation-seeking questions are a type of higher-level discourse that can promote active learning and the development of higher-order thinking skills in students. Second, we demonstrated the essential role of extended questions in promoting students’ systems thinking. One major distinction between leaders and regular students is leader students continuously link multiple sources of information, look for discrepancies and ask sustainable questions, whereas regular students terminate an inquiry thread by judging their peers’ ideas. This pattern is also observed in knowledge-building discourse in which students continuously improved community knowledge through asking sustainable questions (Scardamalia, 2002).

### Implications

Methodologically, this study follows the paradigm of using network analysis approach to analyze students’ engagement in asynchronous forums (Liu et al., 2021, 2022). It also extends prior methods of integrating SNA and ENA beyond its original preservice teacher training context (Ouyang et al., 2021; Ouyang and Dai, 2022). We showed a preliminary understanding of how different groups of students participated in the discussions by analyzing the network and centrality metrics. We then used ENA to understand the participation characteristics of each group. The corroborated findings from both types of analyses showed the nuanced differences in participation patterns from different groups which provide an understanding of what types of participation norms might have led to improved systems thinking.

Practically, this study shed light on future educational practices, especially on how to support online discussions, especially during the pandemic. First, we showed the effectiveness of allowing students to initiate questions and engaging students in follow-up responses in developing systems thinking. With less sophisticated technological tools, this study shows the feasibility of using asynchronous forums in promoting students’ higher-order thinking skills (i.e., systems thinking), which provides a possibility for teachers’ hasty transformation to online teaching during a pandemic. However, teachers should not expect students would engage in high-level online discussions automatically. In this study, the teacher provided on time replies on every post. In addition, teachers could also promote students’ discussions from the following perspectives. First, teachers should encourage students to make broader connections with other students. Meanwhile, the teacher not only should encourage students to ask explanation-seeking questions but also allows students to know how to ask high systems thinking level questions. Rather than providing static scaffolds during the online sessions, the teacher could offer students epistemic training allowing them to know the epistemic criteria of good questions during online synchronous sessions. Second, the teacher should assist students to sustain their inquiry. Teachers could support progressive inquiries by iteratively highlighting inquiry topics that are not discussed thoroughly. Teachers can encourage students to review existing inquiry topics and reflect on the ones that they want to further discuss. It is also helpful for students to revisit their initial questions and purposefully reflect on the extent to which the new information provided by their peers has been of benefit to their understanding. As an engaging method for forming a community of inquiry, instructors can establish this “revisiting” activity as a regular practice.

## Conclusion

This study explores the effectiveness of encouraging students to initiate questions in an asynchronous discussion forum in supporting systems thinking development. Students perceived this learning environment as effective in promoting systems thinking. To further understand if active participation would lead to better systems thinking development, we carried out SNA to assign three participatory roles to students based on their participation levels: leader, regular, and peripheral. Further results that built upon SNA showed that students in the leader group exhibited significantly better systems thinking skills than those in the other two groups. They asked high systems thinking level questions most of which were explanation-seeking questions, made connections between different types of new information, and, most importantly, asked extended questions to sustain the inquiry. Although students in the regular group also asked explanation-seeking questions and made connections among multiple sources of information, they tended to end inquiry threads prematurely with abrupt evaluations.

This study had several limitations. We only had 22 students as our participants due to the small class size in medical schools in China. Although we used a bootstrapping strategy to mitigate this issue, future studies could be undertaken with a larger sample of around 40 students. The intervention was also short, with a 4-week duration. Future studies might consider implementing and evaluating the inquiry-based learning approach in online discussion forums over an extended period. Moreover, the instructor played an essential role in supporting the students’ inquiry process. Due to space constraints her role was not explored in detail. For future studies, we plan to examine not only teachers’ facilitative roles but also how classroom teachers can use analytical tools and pedagogical strategies to support students’ systems thinking development and collective ideas improvements.

## Data availability statement

The raw data supporting the conclusions of this article will be made available by the authors, without undue reservation.

## Ethics statement

The studies involving human participants were reviewed and approved by The University of Hong Kong. The patients/participants provided their written informed consent to participate in this study.

## Author contributions

YY is responsible for analyzing data, framing and writing. GC, YT, and XL is responsible for improving and revising the initial draft. LY provided feedback on analyzing the data. SD was the classroom teacher and collected the data. All authors contributed to the article and approved the submitted version.

## Funding

This work is supported by China National Social Science Fund (Grant No. BCA180095).

## Conflict of interest

The authors declare that the research was conducted in the absence of any commercial or financial relationships that could be construed as a potential conflict of interest.

## Publisher’s note

All claims expressed in this article are solely those of the authors and do not necessarily represent those of their affiliated organizations, or those of the publisher, the editors and the reviewers. Any product that may be evaluated in this article, or claim that may be made by its manufacturer, is not guaranteed or endorsed by the publisher.
